# The ventricular tachycardia prediction model: Derivation and validation data

**DOI:** 10.1016/j.dib.2020.105515

**Published:** 2020-04-21

**Authors:** Anthony H. Kashou, Christopher V. DeSimone, David O. Hodge, Rickey Carter, Grace Lin, Samuel J. Asirvatham, Peter A. Noseworthy, Abhishek J. Deshmukh, Adam M. May

**Affiliations:** a Department of Medicine, Mayo Clinic, United States; b Department of Cardiovascular Diseases, Mayo Clinic, United States; c Department of Department of Health Sciences Research, Mayo Clinic, United States; d Department of Medicine, Division of Cardiovascular Diseases, Washington University in St. Louis, United States

**Keywords:** Electrocardiogram, Wide complex tachycardia, Ventricular tachycardia, Supraventricular tachycardia, Computerized electrocardiogram interpretation

## Abstract

In a recent publication [1], we introduced and described a novel means (i.e. VT Prediction Model) to correctly categorize wide complex tachycardias (WCTs) into ventricular tachycardia (VT) and supraventricular wide complex tachycardia (SWCT) using routine measurements shown on electrocardiogram (ECG) paper recordings. In this article, we summarize data components relating to the derivation and validation of the VT Prediction Model.

Specifications table

**Table:** 

Subject area	Cardiology
More specific subject area	Electrocardiology, computerized electrocardiogram interpretation
Type of data	Tables, figures, and images
How data was acquired	Review of health records and automated measurements provided by computerized electrocardiogram interpretation software (MUSE by *GE Healthcare;* Milwaukee, WI)
Data format	Raw and analyzed data
Parameters for data collection	Evaluated electrocardiograms were paired wide complex tachycardia and baseline electrocardiograms attained within clinical settings throughout the entire Mayo Clinic enterprise between September 2011 and December 2018.
Description of data collection	Evaluated electrocardiograms were standard 12-lead recordings obtained from Mayo Clinic's centralized electrocardiogram data archives. Wide complex tachycardias were required to fulfill wide complex tachycardia criteria (QRS duration ≥ 120 ms; heart rate ≥ 100 bpm) plus a formal ECG laboratory interpretation of (i) "ventricular tachycardia," (ii) "supraventricular tachycardia," or (iii) "wide complex tachycardia." Baseline electrocardiograms were either the first subsequent electrocardiogram or most proximate that did not fulfill wide complex tachycardia criteria.
Data source location	Mayo Clinic
Data accessibility	Data is included in this article
Related research article	A.M. May, C.V. DeSimone, A.H. Kashou, H. Sridhar, D.O. Hodge, R. Carter, G. Lin, S.J. Asirvatham, P.A. Noseworthy, A.J. Deshmukh. The VT Prediction Model: A Simplified Means to Differentiate Wide Complex Tachycardias. Journal of Cardiovascular Electrophysiology. December 2019. 10.1111/jce.14321.

## Value of the data

• Enclosed data summarizes the patient demographics, clinical features, and ECG laboratory interpretation codes of patient cohorts used to derive and validate a novel WCT differentiation method known as the VT Prediction Model. Featured data also describes electrocardiographic characteristics of WCTs accurately and erroneously classified by the VT Prediction Model.
• Data would be valuable to researchers wanting to understand the patient demographics, clinical characteristics, and electrocardiographic features of WCT events customarily encountered in general clinical practice.
• Data would be of value to researchers aiming to specify clinical and ECG features to be examined in prospective evaluations that compare the diagnostic performance of WCT differentiation algorithms.

## 1 Data description

Table 1 summarizes the clinical and ECG laboratory diagnosis data for the derivation cohort. Heart rhythm or non-heart rhythm cardiologists were responsible for most (85.6%) clinical diagnoses. The ECG laboratory assigned definitive or probable interpretive diagnoses to a sizeable majority (94.8%) of WCTs. A minority of evaluated WCTs (32.1%) were derived from patients who underwent an electrophysiology procedure. A sizeable proportion of evaluated WCTs (38.2%) was derived from patients who possessed an implantable intra-cardiac device (e.g., pacemaker).

**Table 1:** Derivation Cohort: clinical and ECG laboratory diagnosis.

	SWCT (*n* = 328)	VT (*n* = 273)	P-Value
Diagnosing provider			
Heart rhythm cardiologists	141 (43.0)	248 (90.8)	
Non-heart rhythm cardiologists	109 (33.2)	17 (6.2)	< 0.0001
Non-cardiologists	78 (23.8)	8 (2.9)	
ECG lab interpretation			
Definite VT	10 (3.0)	226 (82.8)	< 0.0001
Probable VT	16 (4.9)	26 (9.5)	
Definite SWCT	265 (80.8)	6 (2.2)	
Probable SWCT	16 (4.9)	5 (1.8)	
Undifferentiated	21 (6.4)	10 (3.7)	
Time separation between WCT and baseline ECG (hours)			
Mean (SD)	381.7 (2183.2)	160.2 (632.4)	0.77
Median	6.3	8.1	
Q1, Q3	1.0, 43.1	1.1, 46.3	
Time separation between WCT and baseline ECG			
< 3 h	134 (40.9)	111 (40.8)	0.04
3 - 24 h	88 (26.8)	63 (23.1)	
24 h - 30 days	78 (23.8)	87 (319)	
> 30 days	28 (8.5)	12 (4.4)	
Electrophysiology procedure			
Yes	51 (15.5)	142 (52.0)	< 0.0001
Implanted Device			
Yes	49 (14.9)	181 (66.3)	< 0.0001

Numbers in parentheses are percent (%) of n or standard deviation. SD = standard deviation; SWCT = supraventricular tachycardia; VT = ventricular tachycardia.

Table 2 describes the patient characteristics of the derivation cohort. The VT group included more ECG pairs from patients with coronary artery disease, prior myocardial infarction, ischemic cardiomyopathy, non-ischemic cardiomyopathy, active antiarrhythmic drug use, and implanted cardioverter-defibrillator. The supraventricular wide complex tachycardia (SWCT) group comprised more patients having an implanted pacemaker. Baseline ECGs demonstrating ventricular pacing were more prevalent in the ventricular tachycardia (VT) group than the SWCT group. Baseline bundle branch block was more common in the SWCT group than the VT group. No SWCTs (0.0%) demonstrated pre-excitation.

**Table 2:** Derivation cohort: patient characteristics.

	SWCT (*n* = 328)	VT (*n* = 273)	P-Value
Age (years)			
Mean (SD)	70.6 (14.6)	65.8 (13.1)	< 0.0001
Range	18.0 - 98.0	27.0 - 90.0	
Gender			
Male	212 (64.6)	225 (82.4)	< 0.0001
Female	116 (35.4)	48 (17.6)	
Clinical Characteristics			
Coronary artery disease	160 (48.8)	188 (68.9)	< 0.0001
Prior myocardial infarction	93 (28.4)	157 (57.5)	< 0.0001
Prior heart surgery	123 (37.5)	118 (43.2)	0.15
Congenital heart disease	18 (5.5)	19 (7.0)	0.45
Anti-arrhythmic drug use	52 (15.9)	165 (60.4)	< 0.0001
Ischemic cardiomyopathy	52 (15.9)	138 (50.5)	< 0.0001
Non-ischemic cardiomyopathy	77 (23.5)	89 (32.6)	0.01
AICD	22 (6.7)	176 (64.5)	< 0.0001
Pacemaker	27 (8.2)	5 (1.8)	0.0005
Left ventricular ejection fraction (%)			
Unknown LVEF	14 (4.3)	1 (0.4)	< 0.0001
LVEF (<= 30)	66 (20.1)	118 (43.2)	
LVEF (49 - 31)	59 (18.0)	85 (31.1)	
LVEF (>= 50)	189 (57.6)	69 (25.3)	
Baseline ECG			
Baseline bundle branch block	217 (66.2)	39 (14.3)	< 0.0001
Baseline ventricular pacing	19 (5.8)	110 (40.3)	< 0.0001
SWCT with pre-excitation			
Yes	0 (0.0%)	***	***

Numbers in parentheses are percent (%) of n or standard deviation. AICD = automatic implantable cardioverter-defibrillator; LVEF = left ventricular ejection fraction; SD = standard deviation; SWCT = supraventricular tachycardia; VT = ventricular tachycardia.

Table 3 summarizes the clinical and ECG laboratory diagnosis data for the validation cohort. Heart rhythm or non-heart rhythm cardiologists were responsible for most (90.5%) clinical diagnoses. Definitive or probable diagnoses were assigned to the vast majority (95.0%) of WCTs interpreted by the ECG laboratory. About one-third (34.4%) of evaluated WCTs were derived from patients who underwent an electrophysiology procedure. A substantial percentage (40.2%) of evaluated WCTs were derived from patients who possessed an implantable intra-cardiac device.

**Table 3:** Validation cohort: clinical and ECG laboratory diagnosis.

	SWCT (*n* = 144)	VT (*n* = 97)	P-Value
Diagnosing provider			
Heart rhythm cardiologists	59 (41.0)	82 (84.5)	< 0.001
Non-heart rhythm cardiologists	63 (43.8)	14 (14.4)	
Non-cardiologists	22 (15.3)	1 (1.0)	
ECG lab interpretation			
Definite VT	9 (6.2)	68 (70.1)	< 0.001
Probable VT	1 (0.7)	9 (9.3)	
Definite SWCT	87 (60.4)	3 (3.1)	
Probable SWCT	41 (28.5)	11 (11.3)	
Undifferentiated	6 (4.2)	6 (6.2)	
Time separation between WCT and baseline ECG (hours)			
Mean (SD)	275.9 (1293.8)	104.3 (434.8)	0.075
Median	10.1	5.1	
Q1, Q3	1.5, 46.6	0.6, 33.8	
Time separation between WCT and Baseline ECG			
< 3 h	47 (32.6)	45 (46.4)	0.174
3 - 24 h	46 (31.9)	23 (23.7)	
24 h - 30 days	46 (31.9)	27 (27.8)	
> 30 days	5 (3.5)	2 (2.1)	
Electrophysiology procedure			
Yes	26 (18.1)	57 (58.8)	< 0.001
Implanted device			
Yes	27 (18.8)	70 (72.2)	< 0.001

Numbers in parentheses are percent (%) of n or standard deviation. SD = standard deviation; SWCT = supraventricular tachycardia; VT = ventricular tachycardia.

Table 4 details the clinical characteristics of patients comprising the validation cohort. The VT group included more ECG pairs from patients with coronary artery disease, prior myocardial infarction, ischemic cardiomyopathy, active antiarrhythmic drug use, and implanted cardioverter-defibrillator. The SWCT group comprised more ECG pairs from patients having an implanted pacemaker. Baseline ECGs demonstrating ventricular pacing were more prevalent in the VT group than the SWCT group. Baseline bundle branch block was more common in the SWCT group than the VT group. Four SWCTs (2.8%) demonstrated pre-excitation.

**Table 4:** Validation cohort: clinical and ECG laboratory diagnosis.

	SWCT (*n* = 144)	VT (*n* = 97)	P-Value
Age (years)			
Mean (SD)	69.4 (14.8)	67.5 (10.8)	0.027
Gender			
Male	102 (70.8)	87 (89.7)	< 0.001
Female	42 (29.2)	10 (10.3)	
Clinical Characteristics			
Coronary artery disease	54 (37.5)	71 (73.2)	< 0.001
Prior myocardial infarction	34 (23.6)	67 (69.1)	< 0.001
Prior heart surgery	45 (31.2)	29 (29.9)	0.823
Congenital heart disease	5 (3.5)	0 (0.0)	0.064
Anti-arrhythmic drug use	25 (17.4)	48 (49.5)	< 0.001
Ischemic cardiomyopathy	27 (18.8)	63 (64.9)	< 0.001
Non-ischemic cardiomyopathy	49 (34.0)	24 (24.7)	0.124
AICD	21 (14.6)	70 (72.2)	< 0.001
Pacemaker	7 (4.9)	0 (0.0)	0.028
Left ventricular ejection fraction (%)			
Unknown LVEF	7 (4.9)	1 (1.0)	< 0.001
LVEF (<= 30)	35 (24.3)	45 (46.4)	
LVEF (49 - 31)	27 (18.8)	31 (32.0)	
LVEF (>= 50)	75 (52.1)	20 (20.6)	
Baseline ECG			
Baseline bundle branch block	90 (62.5)	17 (17.5)	< 0.001
Baseline ventricular pacing	8 (5.6)	33 (34.0)	< 0.001
SWCT with Pre-excitation			
Yes	4 (2.8)	***	***

Numbers in parentheses are percent (%) of n or standard deviation. AICD = automatic implantable cardioverter-defibrillator; LVEF = left ventricular ejection fraction; SD = standard deviation; SWCT = supraventricular tachycardia; VT = ventricular tachycardia.

Table 5 summarizes electrocardiographic characteristics of correct and incorrect diagnoses established by the VT Prediction Model for the derivation cohort. According to a 50% VT probability partition to establish VT diagnoses (VT > = 50.0% and SWCT < 50.0%), 53 out of 278 (19.1%) clinical VTs were incorrectly branded as SWCT by the VT Prediction Model. In comparison with correctly identified VTs, erroneous classifications of clinical VT as SWCT displayed shorter WCT QRS duration and constrained changes in QRS duration, QRS axis, and T axis between paired baseline and WCT ECGs. According to a 50% VT probability partition to establish VT diagnoses (VT > = 50.0% and SWCT < 50.0%), 38 out of 323 (11.8%) clinical SWCTs were erroneously categorized as VT by the VT Prediction Model. In comparison with correctly identified SWCTs, erroneous classifications of clinical SWCT as VT demonstrated more prolonged WCT QRS intervals and larger changes in QRS duration, QRS axis, and T axis between paired baseline and WCT ECGs.

**Table 5:** Derivation cohort: correct and erroneous WCT diagnoses.

	WCT (*n* = 601)					
	VT (*n* = 273)			SWCT (*n* = 328)		
	Erroneous SWCT Prediction (*n* = 53)	Correct VT Prediction (*n* = 220)	P-value	Erroneous VT Prediction (*n* = 38)	Correct SWCT Prediction (*n* = 290)	P-value
WCT QRS duration (ms)	147.7 (19.7)	183.9 (30.3)	< 0.0001	163.6 (20.1)	140.3.6 (15.7)	< 0.0001
QRS duration change (ms)	24.7 (17.9)	51.5 (35.9)	< 0.0001	41.5 (33.6)	13.7 (14.2)	< 0.0001
QRS axis change (°)	35.2 (36.5)	99.0 (54.8)	< 0.0001	68.7 (56.9)	20.1 (23.7)	< 0.0001
T axis change (°)	50.1 (46.4)	101.8 (55.8)	< 0.0001	92.9 (47.8)	34.1 (35.1)	< 0.0001

Displayed numbers represent mean values. Numbers in parentheses are standard deviation. The erroneous VT prediction group comprise clinical SWCTs assigned high VT probability (>= 50%). The erroneous SWCT prediction group comprise clinical VTs assigned low VT probability (< 50%). SWCT = supraventricular wide complex tachycardia; VT= ventricular tachycardia; WCT = wide complex tachycardia.

**Fig. 1:**
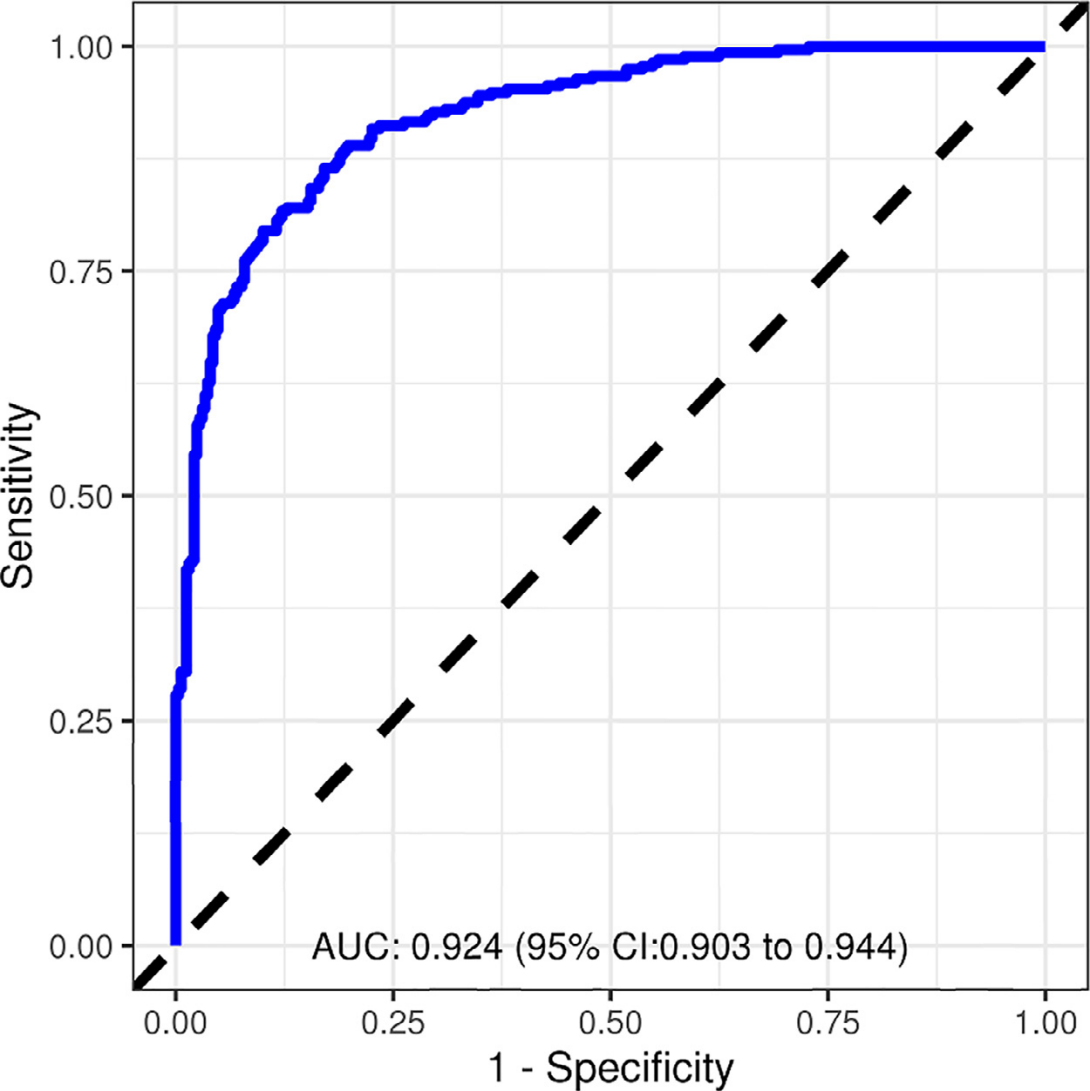
VT prediction model diagnostic performance: derivation cohort.

Fig. 1 illustrates the diagnostic performance of the VT Prediction Model for the derivation cohort (AUC 0.924; CI 0.903 – 0.944).

**Fig. 2:**
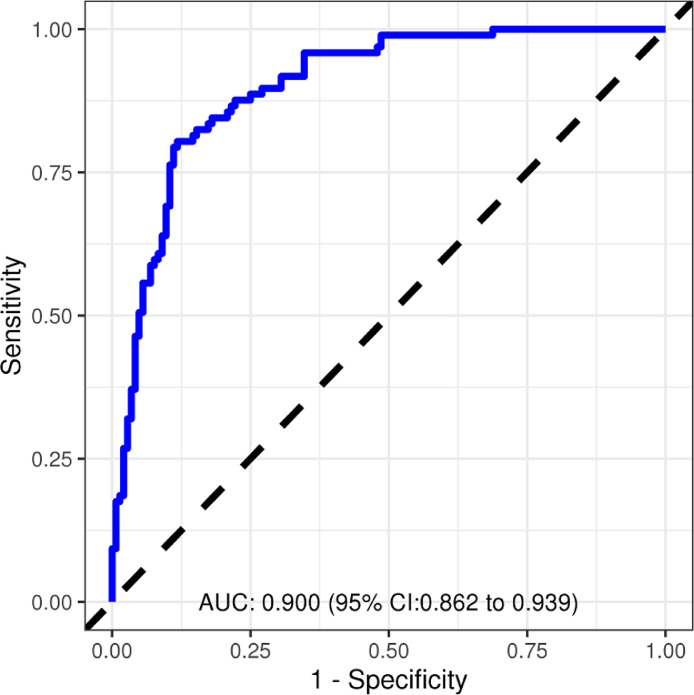
VT prediction model diagnostic performance: validation cohort.

Fig. 2 illustrates the diagnostic performance of the VT Prediction Model when implemented on the validation cohort (AUC 0.900; CI 0.862 – 0.939).

**Fig. 3:**
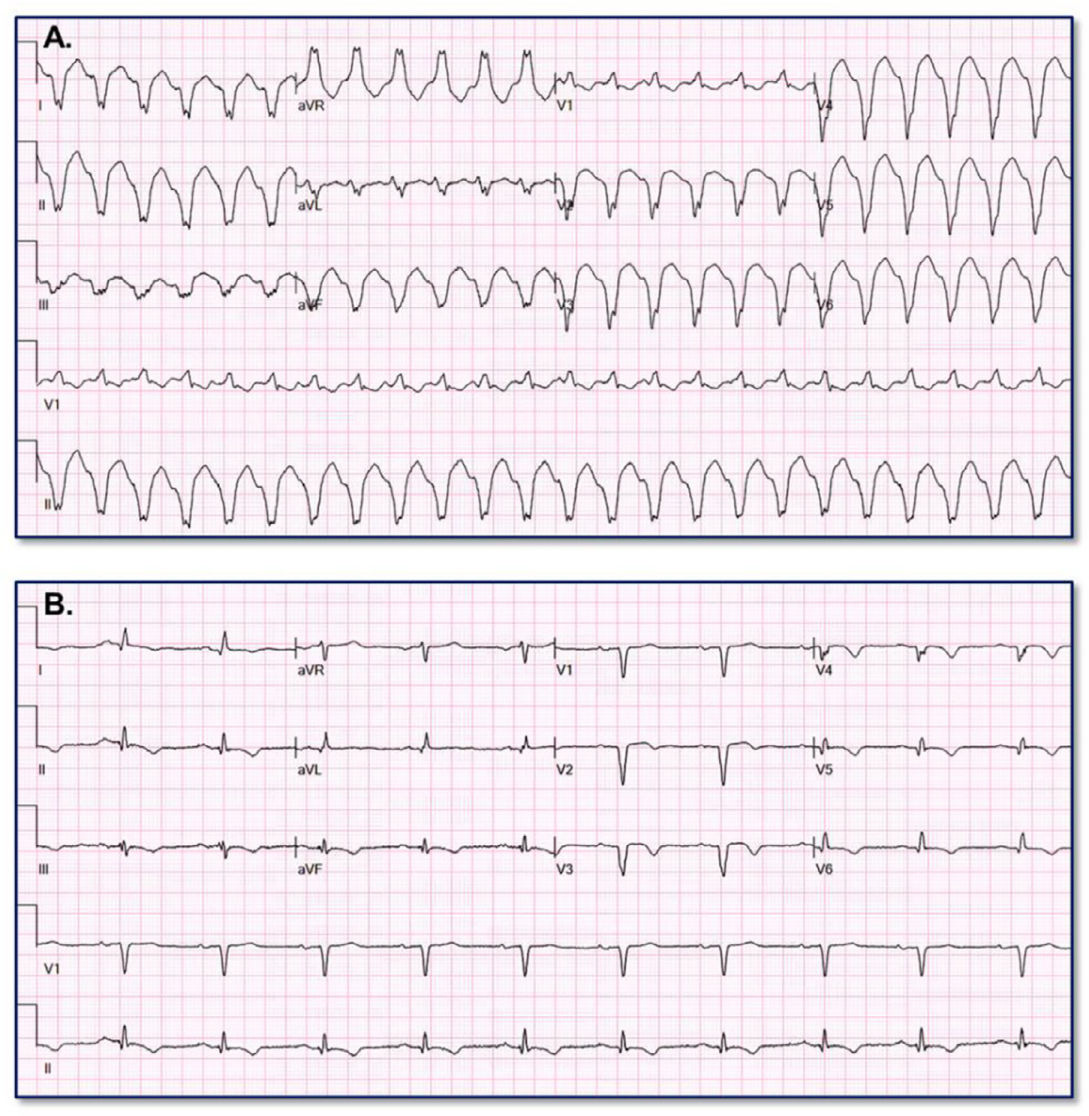
Appropriate high VT probability assignment.

Fig. 3 provides an example of paired VT (A) and baseline (B) ECGs assigned high VT probability (99.0006%) by the VT Prediction Model. WCT QRS duration = 182 ms; QRS duration change = 48 ms; QRS axis change = 134°; T axis change = 93°

**Fig. 4:**
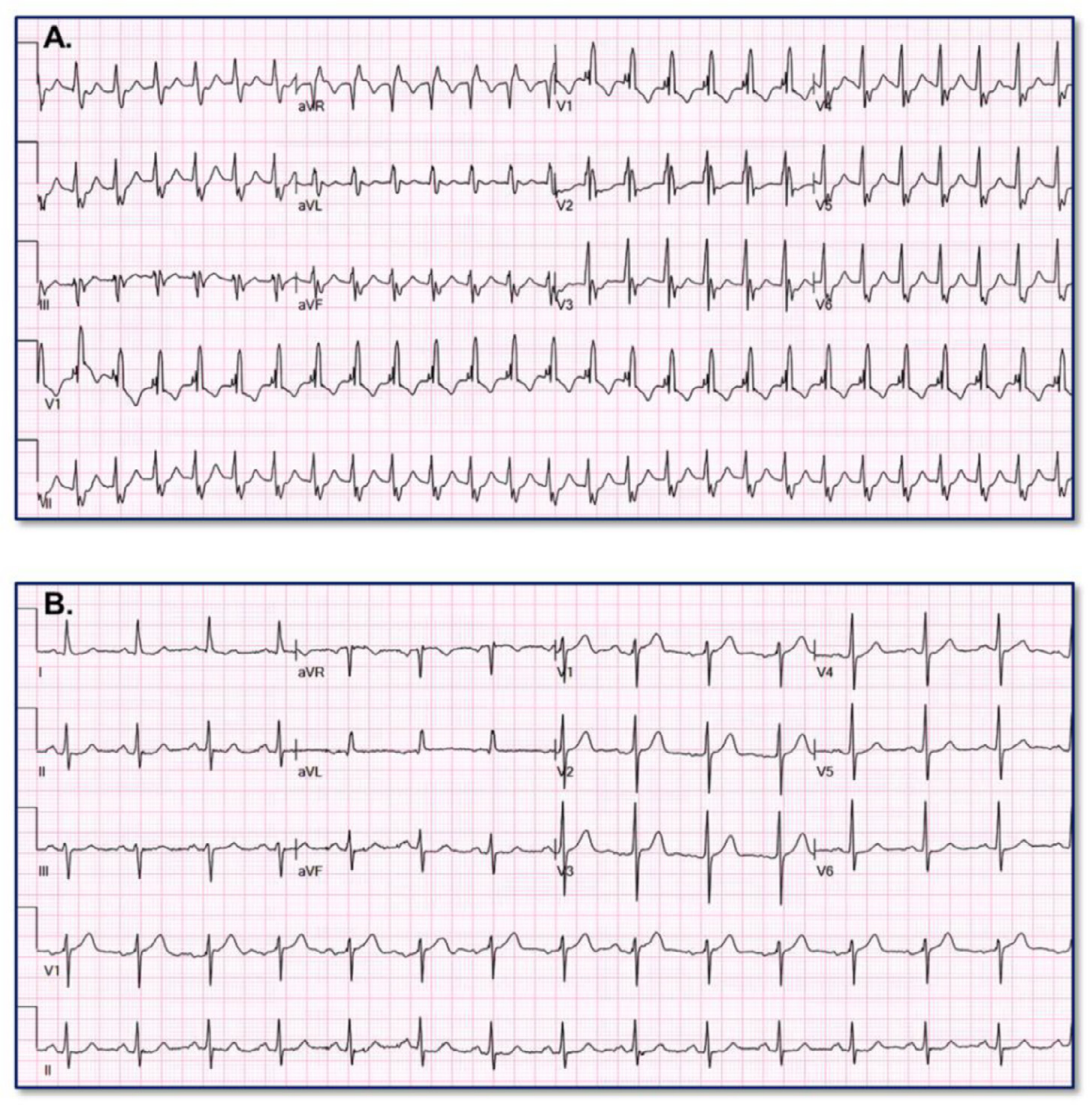
Appropriate low VT probability assignment.

Fig. 4 provides an example of paired SWCT (A) and baseline (B) ECGs assigned low VT probability (4.3609%) by the VT Prediction Model. WCT QRS duration = 130 ms; QRS duration change = 46 ms; QRS axis change = 1°; T axis change = 8°

**Fig. 5:**
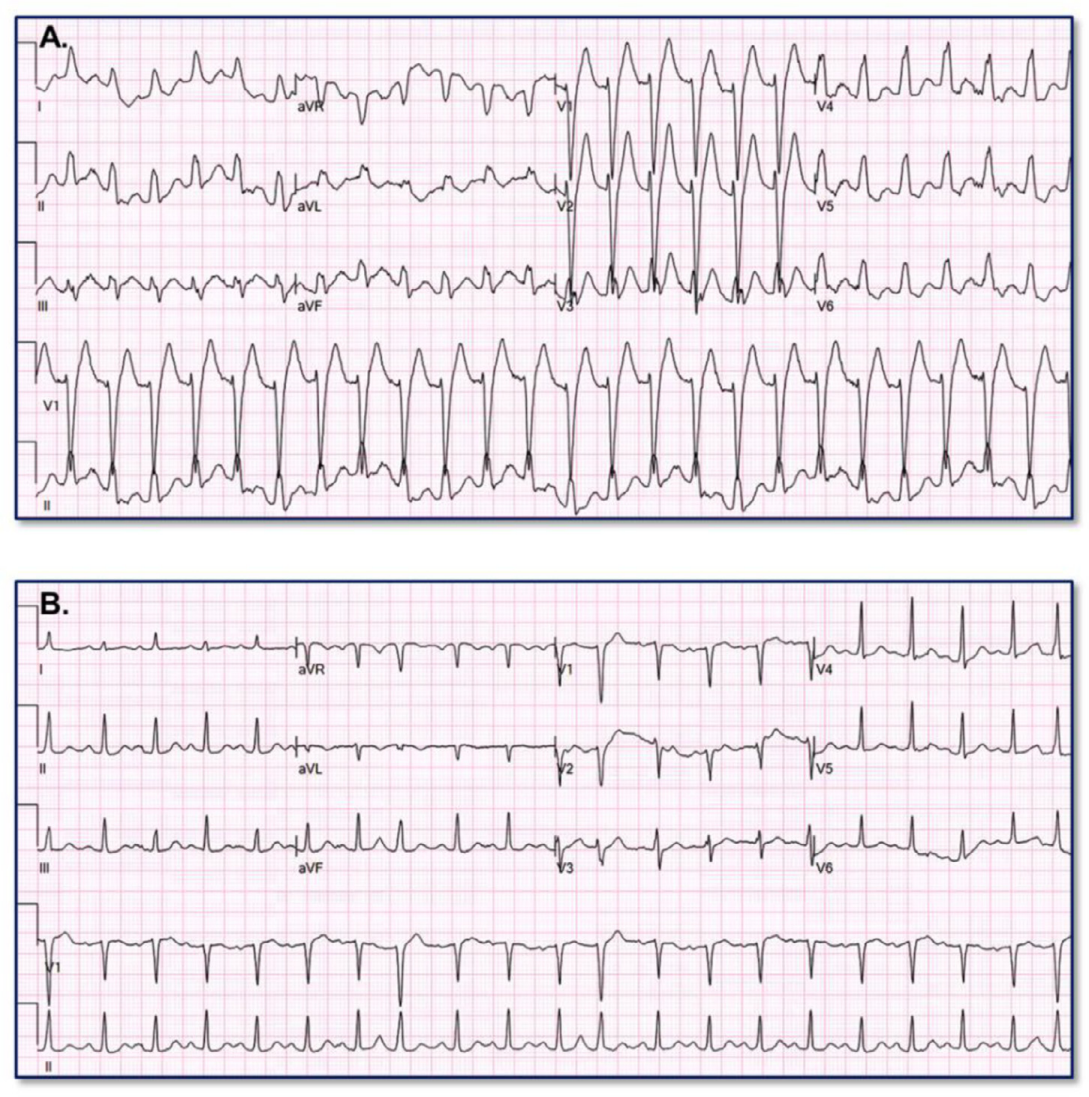
Appropriate low VT probability assignment.

Fig. 5 provides an example of paired SWCT (A) and baseline (B) ECGs assigned low VT probability (6.3613%) by the VT Prediction Model. WCT QRS duration = 120 ms; QRS duration change = 36 ms; QRS axis change = 48°; change T axis change = 13°

**Fig. 6:**
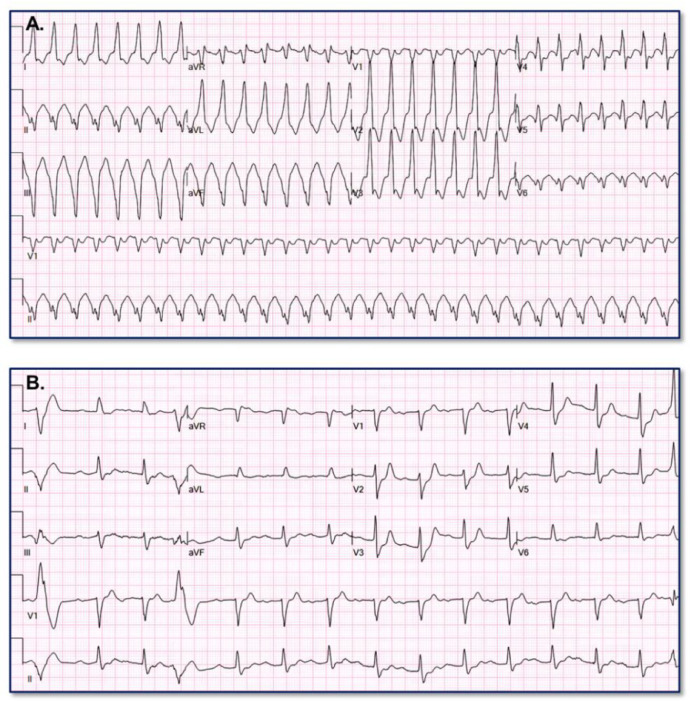
Erroneous low VT probability assignment.

Fig. 6 provides an example of paired VT (A) and baseline (B) ECGs assigned low VT probability (9.8704%) by the VT Prediction Model. WCT QRS duration = 126 ms; QRS duration change = 6 ms; QRS axis change = 63°; T axis change = 30°

**Fig. 7:**
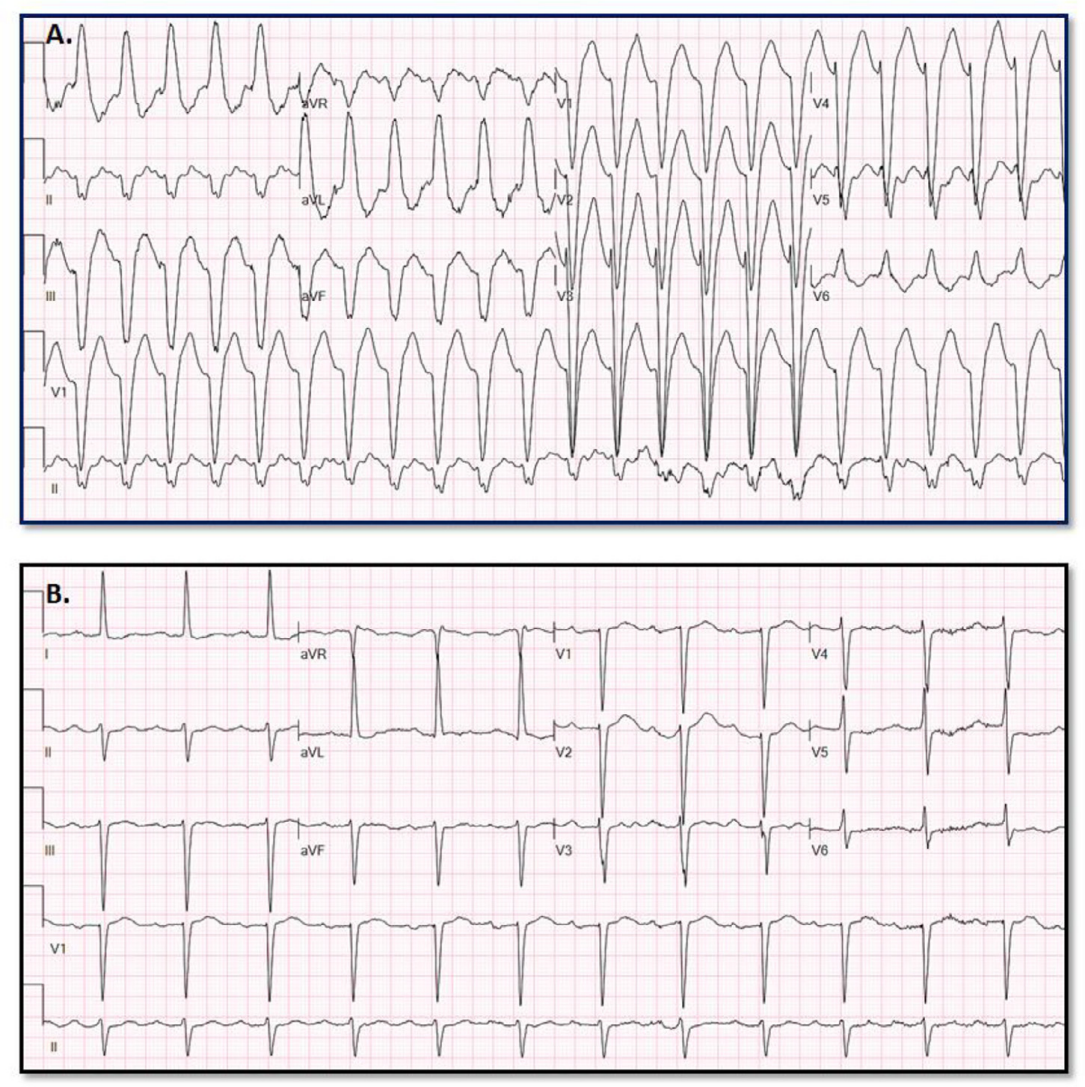
Erroneous high VT probability assignment.

Fig. 7 provides an example of paired SWCT (A) and baseline (B) ECGs assigned high VT probability (54.0039%) by the VT Prediction Model. WCT QRS duration = 170 ms; QRS duration change = 52 ms; QRS axis change = 3°; T axis change = 89°

## 2 Experimental design, materials, and methods

Our recent publication [1] describes the derivation and implementation of a new WCT differentiation method that produces explicit VT probability estimations for paired WCT and baseline ECGs. In this report, a logistic regression model (i.e., VT Prediction Model) was derived and tested using two using separate patient cohorts: derivation and validation. First, a derivation cohort of paired WCT and baseline ECGs was evaluated to identify independent predictors to be consolidated into the VT Prediction Model. After that, the VT Prediction Model was trialed against a separate validation cohort of paired WCT and baseline ECGs. The overall diagnostic performance of the VT Prediction Model was appraised according to its agreement with the final clinical diagnosis established by patients' supervising physicians.

Paired WCT and baseline ECGs were derived from actual clinical settings throughout the entire Mayo Clinic enterprise between September 2011 and December 2018. Evaluated ECGs were standard 12-lead paper recordings (speed: 25 mm/s, voltage calibration: 10 mm/mV) acquired from Mayo Clinic's ECG data archives (*GE Healthcare;* Milwaukee, WI).

Included WCTs were required to fulfill WCT criteria (QRS duration ≥ 120 ms and heart rate ≥ 100 bpm) plus an official ECG laboratory interpretation of (1) "ventricular tachycardia," (2) "supraventricular tachycardia," or (3) "wide complex tachycardia." Baseline ECGs were either the first subsequent ECG (i.e., for the derivation cohort) or nearest ECG (i.e., for the validation cohort), not fulfilling WCT criteria.

The derivation cohort encompassed 601 paired WCT (273 VT, 328 SWCT) and baseline ECGs from 421 patients presenting to Mayo Clinic Rochester or Mayo Clinic Health System of South Eastern Minnesota (September 2011 through November 2016). The validation cohort comprised 241 WCT (97 VT, 144 SWCT) and baseline ECG pairs from 177 patients presenting to the whole Mayo Clinic enterprise (January 2018 through December 2018) – including three large medical centers (Rochester, Minnesota; Jacksonville, Florida; and Phoenix/Scottsdale, Arizona) and auxiliary patient care locations (e.g., community hospitals).

Data relating to clinical diagnosis, ECG laboratory examination, and patient characteristics were discovered from an electronic medical record review. Standard ECG measurements rendered by *GE Healthcare's* MUSE ECG interpretation software were acquired from archived ECG recordings. Basic arithmetical computations (QRS axis change, T wave axis change, QRS duration change) were processed using electronic measurements routinely displayed on ECG recordings.

The Mayo Clinic Institutional Review Board approved patient data acquisition and analysis. Similar patient selection processes and data reporting were previously adopted in a separate analysis [2,3].

## Appendix Supplementary materials

Image, application 1

Image, application 2

Image, application 3
